# A 40-Class SSVEP Speller Dataset: Beta Range Stimulation for Low-Fatigue BCI Applications

**DOI:** 10.1038/s41597-025-06032-2

**Published:** 2025-11-05

**Authors:** Heegyu Kim, Kyungho Won, Minkyu Ahn, Sung Chan Jun

**Affiliations:** 1 School of Electrical Engineering and Computer Science, Gwangju Institute of Science and Technology, Gwangju, South Korea; 2 Department of Embedded System Engineering, Incheon National University, Incheon, South Korea; 3 School of Computer Science and Electrical Engineering, Handong Global University, Pohang, South Korea; 4 AI Graduate School, Gwangju Institute of Science and Technology, Gwangju, South Korea

**Keywords:** Cognitive control, Brain-machine interface

## Abstract

The inherent non-stationarity of electroencephalography (EEG) signals necessitates large, consistent datasets for reliable brain–computer interface (BCI) research. In steady-state visual evoked potential (SSVEP) paradigms, prolonged exposure to visual stimuli can induce visual fatigue, leading to alterations in EEG patterns that degrade BCI performance. To mitigate fatigue-induced variability, this study employs visual stimulation in the beta frequency range (14–22 Hz), a range that appears less susceptible to the effects of fatigue. We present a comprehensive 40-class SSVEP speller dataset acquired from 40 participants, with EEG data recorded from 31 central-to-occipital channels. Each subject completed six sessions of the SSVEP speller task in addition to pre- and post-experiment resting-state recordings under both eyes-open and eyes-closed conditions. Subjective fatigue ratings combined with EEG band power analyses confirm that beta-range stimulation minimizes fatigue effects. Moreover, the high classification accuracy achieved by calibration-based algorithms indicates that the dataset is well-suited for training advanced SSVEP-based BCI systems.

## Background & Summary

The Brain–Computer Interface (BCI) is a technology that facilitates communication with external devices by decoding brain activity and transmitting commands^1–3^. BCI technology is being developed for clinical applications such as virtual keyboards, wheelchairs, and rehabilitation systems for patients with severe motor or communication impairments^4^. It is also being explored for everyday applications, including virtual reality and smart home control^5^.

The Steady-State Visual Evoked Potential (SSVEP) is a brain signal characterized by specific frequency components elicited by periodic visual stimuli^6^. Due to its high signal-to-noise ratio (SNR) and information transfer rate (ITR), SSVEP is widely used in BCI paradigms, allowing for the modulation of multiple commands via visual targets^7^. Typically, the high-speed performance of BCIs is achieved through multi-target visual spellers. The term “BCI illiteracy” describes the difficulty some individuals experience in effectively using BCI paradigms; however, the user-friendly design of SSVEP systems and their low incidence of BCI illiteracy make them promising candidates for real-world applications^8^.

Significant advancements have been made to enhance BCI performance in SSVEP frequency recognition^9^. Owing to its inherent properties, SSVEP can achieve high classification performance even with zero-training recognition methods such as canonical correlation analysis (CCA) and filter bank CCA (FB-CCA). Nevertheless, further improvements have been demonstrated using individual template (IT)-CCA, task-related component analysis (TRCA), and various machine learning techniques^10^. Recently, there has been an increase in attempts to use deep learning methods—including convolutional neural networks (CNNs), long short-term memory networks (LSTMs), Transformers, and generative adversarial networks (GANs)—for SSVEP classification^8^. To develop generalizable BCI systems, subject-independent classifiers are needed, which require data from multiple subjects.

In response to these requirements, numerous SSVEP BCI datasets have been made publicly available. Several notable datasets feature multi-class SSVEP speller data utilizing target frequencies within the alpha range (8–16 Hz)^11,12^ as well as SSVEP paradigms that include four directional commands^13,14^. Moreover, there are datasets that focus on SSVEP speller frequencies between 9 and 14 Hz, alongside those that span a wider frequency range (1–60 Hz) to explore the general characteristics of SSVEP^15,16^.

Due to the inherent exposure to periodic visual stimuli, SSVEP paradigms often induce visual fatigue, which can lead to significant alterations in EEG signals and compromise classification performance^17,18^. Studies have reported that visual fatigue particularly affects brain activity in the occipital region, causing increases in delta, theta, and alpha power, while beta and gamma bands tend to remain relatively stable^19^. Given the nonstationary nature of EEG signals, reducing such variability is crucial for improving model training and classification accuracy^20,21^. Fatigue can also reduce the signal-to-noise ratio (SNR) of SSVEP responses and impair individual calibration accuracy^22,23^. In contrast to alpha and theta bands, where amplitude fluctuations occur over time, the beta range exhibits more consistent characteristics. Prior research has shown that while alpha power increases and theta activity decreases under fatigue, beta band activity remains relatively unaffected^24^. Consistent with these findings, our results also showed notable increases in alpha power with minimal changes in the beta band.

A standard method for estimating visual fatigue in EEG analysis involves spectral power comparisons^19,25^, particularly focusing on the absolute and relative power increases in the alpha and theta bands, which are well-known indicators of fatigue. To quantify visual fatigue, we used the CCA coefficient, which is frequently employed for both qualitative and quantitative assessments of EEG-based fatigue^26^. Beyond its application in the SSVEP paradigm, CCA is widely used for the objective detection of various types of visual fatigue, enabling straightforward and objective quantitative analyses of test data. Furthermore, SSVEP responses typically show a reduction in amplitude due to visual fatigue^27^, with fatigue effects manifesting as changes in frequency peak amplitude or variations in the peak response’s signal-to-noise ratio (SNR)^28^.

This study aims to collect EEG data from a large number of subjects using a massive-class SSVEP-based BCI speller. By employing stimuli within the beta wave range, which is less susceptible to visual fatigue, we aim to reduce inter-subject variability in EEG data. This dataset is designed to support deep learning applications by providing EEG data with reduced variability and improved suitability for training.

## Methods

### Participants

**Fig. 1:**
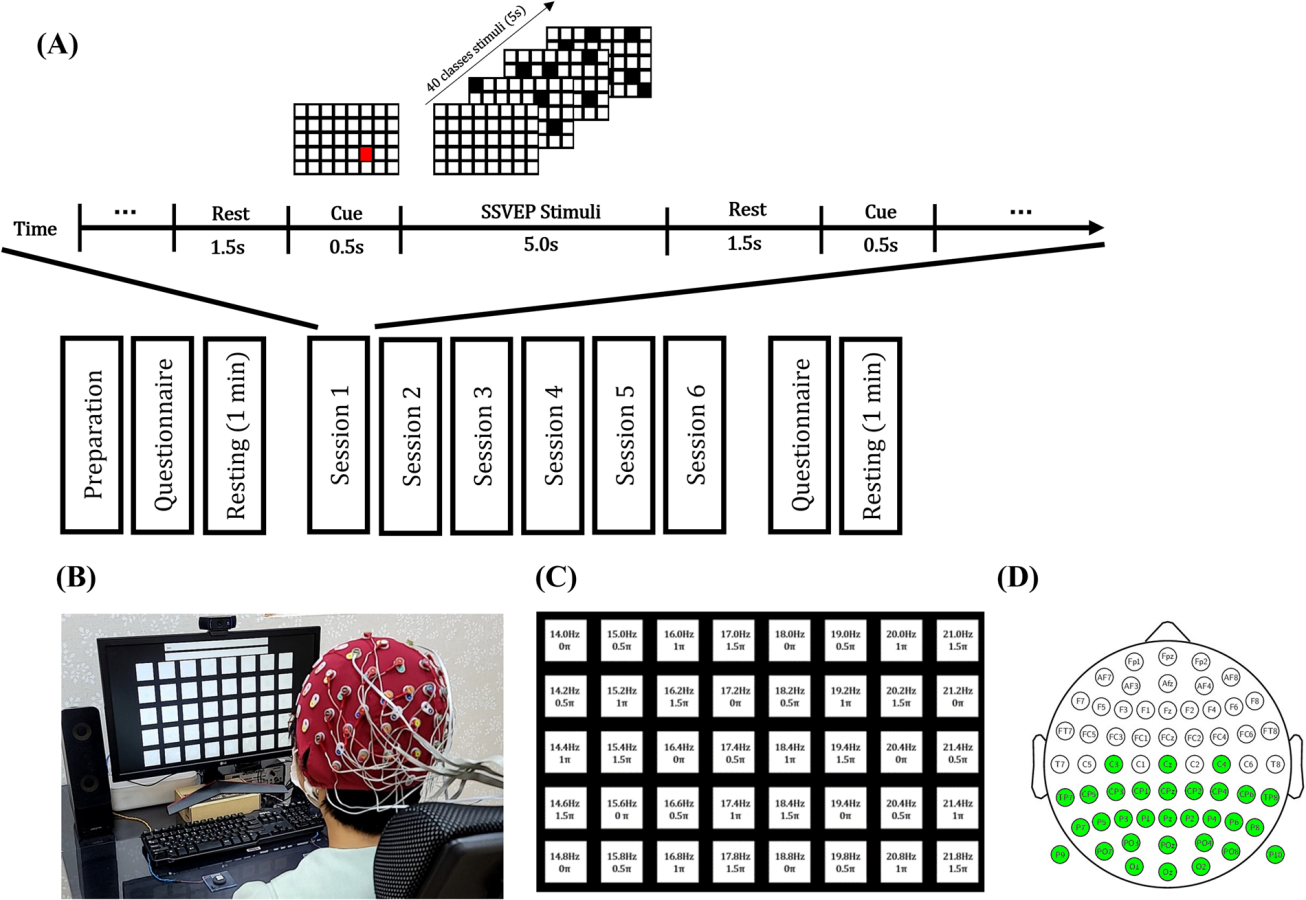
The SSVEP Experimental Paradigm. (**A**) Experiment Timeline: The study included pre- and post-surveys and a resting-state measurement lasting over one minute. It comprised six sessions, each presenting the 40-class stimuli in a randomized order, with each stimulus shown once per session in single day and single study. (**B**) Experimental setup: Participants sat approximately 70 cm away from a monitor while wearing EEG equipment during the experiment. (C) SSVEP Speller Design: The SSVEP speller was organized as an 5 × 8 matrix utilizing JFPM. (D) Electrode Configuration: The research utilized a Biosemi 64-channel system, collecting data from the 31 channels highlighted in green, along with both mastoid electrodes.

A total of 40 subjects (25 males/15 females, with a mean age of 22.8 ± 3.34 years, ranging from 20 to 35 years) participated in the experiment. Among these, three had previous experience with SSVEP-based BCI experiments. Importantly, none of the participants reported any history of neurological, psychiatric, or other conditions that could potentially affect the outcomes. All subjects were positioned at a distance of 70 cm from the monitor during the experiment (as Fig. 1B). The Institutional Review Board at Gwangju Institute of Science and Technology (GIST) approved this experiment (20211201-HR-64-02-04), and all subjects were informed of all experimental procedures and signed consent forms that included confidentiality of personal information (excluding questionnaire-related personal data) as well as consent for the use and open sharing of the data. Data sharing follows the appropriate ethical approval procedures to ensure that the privacy of participants is protected.

### SSVEP Paradigm

The SSVEP speller was presented on a monitor (LG Electronics, Inc., LG24GL600F) with a 1920 × 1080 resolution, 24 inches, and a 120 Hz refresh rate. Presentation software was developed in MATLAB (MathWorks, Inc., MA, USA; ver. 2020a) using Psychophysics Toolbox version 3 (PTB-3)^29^. The frequency and phase parameters for the 40 stimuli were designed using the joint frequency and phase modulation (JFPM) approach^11^. Each stimulus target measured 140 × 140 pixels and was arranged in a 5 × 8 matrix, with 50-pixel gaps between targets in both the vertical and horizontal directions. The flickering frequencies ranged from 14.0 Hz to 21.8 Hz, incremented by 0.2 Hz, with a phase difference of 0.5π between adjacent stimuli (refer to Fig. 1C).

The experiment was structured as a cue-based target selection task. It comprised six blocks, each containing 40 trials that randomly presented all stimuli. Each trial began with a blank screen displayed for 1.5 seconds, followed by a 0.5-second target cue, and then a 5-second flickering period (see Fig. 1A). To maintain engagement, each session included a 1- to 2-minute break prior to the commencement of the next session.

In addition, questionnaires were administered both before and after the experiment. Pre-experimental surveys gathered personal and eye-related information, as well as measurements of the subjects’ mental and physiological states (refer to Table 1). Following the surveys, a resting-state recording of over 1 minute was obtained while a black screen with a central fixation cross (+) was displayed.

**Table 1:** Questionnaire prior to experiments.

Index	Question	Answer
**A**	**Personal information**	
A01	Wearing Glasses	O/X
A02	Physical/Mental Illness	O/X
A03	Eye-related Disease	O/X
A04	Regular Use of Eye-related Medication	O/X
A05	History of Eye-related Surgery	O/X
A06	Experience with BCI Experiments	O/X
A07	Experience with SSVEP Experiments	O/X
A08	Preferred Handedness	R/L/both
A09	Gender	M/F
**B**	**Physiological and Psychological condition**	
B01	How many hours did you sleep last night?	less 4, 4~6, 6~7, 7~8, over 8 hour
B02	Did you drink coffee in the past 24 hours?	0, 0~6, 6~12, 12~18, 18~ 24 hour
B03	Did you consume alcohol in the past 24 hours?	0, 0~6, 6~12, 12~18, 18~ 24 hour
B04	Did you smoke in the past 24 hours?	0, 0~6, 6~12, 12~18, 18~ 24 hour
B05	Display Exposure Environment	0~1, 1~2, 2~3, 3~4, over 5 hour
B06	Daily Computer Usage	0~1, 1~2, 2~3, 3~4, over 5 hour
B07	Daily Smartphone Usage	0~1, 1~2, 2~3, 3~4, over 5 hour
**C**	**Questionnaire prior to experiments**	
C01	Condition - Comfort	Scale (1–5), 5: uncomfortable
C02	Condition -Drowsiness	Scale (1–5), 5: drowsiness
C03	Condition - Concentration	Scale (1–5), 5: concentrate
C04	Condition - Physical condition	Scale (1–5), 5: high fatigue
C05	Condition - Mental condition	Scale (1–5), 5: high fatigue
C06	Condition - Eye fatigue - My eyes feel tired.	Scale (1–5), 5: high fatigue
C07	Condition - Eye fatigue - I am blinking a lot.	Scale (1–5), 5: high fatigue
C08	Condition - Eye fatigue - My eyes feel dry.	Scale (1–5), 5: high fatigue
C09	Condition - Eye fatigue - My eyes feel bloodshot.	Scale (1–5), 5: high fatigue
C10	Condition - Eye fatigue - I feel pain around my eyes.	Scale (1–5), 5: high fatigue
**D**	**Questionnaire posterior to experiments**	
D01	Condition - Comfort	Scale (1–5), 5: uncomfortable
D02	Condition -Drowsiness	Scale (1–5), 5: drowsiness
D03	Condition - Concentration	Scale (1–5), 5: concentrate
D04	Condition - Physical condition	Scale (1–5), 5: high fatigue
D05	Condition - Mental condition	Scale (1–5), 5: high fatigue
D06	Condition - Eye fatigue - My eyes feel tired.	Scale (1–5), 5: high fatigue
D07	Condition - Eye fatigue - I am blinking a lot.	Scale (1–5), 5: high fatigue
D08	Condition - Eye fatigue - My eyes feel dry.	Scale (1–5), 5: high fatigue
D09	Condition - Eye fatigue - My eyes feel bloodshot.	Scale (1–5), 5: high fatigue
D10	Condition - Eye fatigue - I feel pain around my eyes.	Scale (1–5), 5: high fatigue
**E**	**Experimental factor**	
E01	Stiffness in the neck	Scale (1–5), 5: high fatigue
E02	Stiffness in the shoulders	Scale (1–5), 5: high fatigue
E03	Stiffness in the lower back	Scale (1–5), 5: high fatigue
E04	Legs felt stiff or fatigued	Scale (1–5), 5: high fatigue
E05	Felt dazed	Scale (1–5), 5: high fatigue
E06	Felt sleepy during the experiment	Scale (1–5), 5: high fatigue
E07	Felt dizziness during the experiment	Scale (1–5), 5: high fatigue
E08	Felt nervous or irritable	Scale (1–5), 5: high fatigue
E09	Were you able to focus well on the experiment?	Well Manipulated (1) to Poorly (5)
E10	Did you feel like you were actively controlling it?	Well Manipulated (1) to Poorly (5)
E11	Duration of one trial	Short (1) to Long (5)
E12	Total experiment duration	Short (1) to Long (5)
E13	Software used for the experiment	Easy (1) to Difficult (5)
E14	Environment required for the experiment	Good (1) to Poor (5)

### Experimental procedure

The experiment was conducted in the order shown in Fig. 1A. Details are as follows: Each participant began the session with a thorough cleaning of the skin around the mastoid areas before the attachment of electrodes. A brief instructional video was shown to familiarize participants with the experimental procedure and the tasks that would follow. Following this, EEG electrodes were affixed, and checks for impedance and signal quality were conducted. The distance between the participant and the monitor was adjusted to approximately 70 cm to ensure uniform visual presentation. Participants then proceeded to complete the initial set of questionnaires (sections A to C). Subsequently, resting-state EEG data were recorded while a fixation cross was displayed on a black screen—first with participants’ eyes open and then with eyes closed. The main experiment consisted of six sessions, each lasting no longer than five minutes. Short breaks of one to three minutes were provided between sessions to mitigate fatigue. After the final SSVEP session, participants completed the remaining questionnaire items (sections D to E). Following a brief one-minute rest period, a second resting-state EEG was recorded under the same conditions as the initial measurement.

## Data Records

### EEG data recording

EEG signals were recorded at a sampling rate of 1024 Hz using 31 Ag-AgCl wet electrodes, with additional electrodes placed on both mastoids for reference. The BioSemi ActiveTwo system (BSM, BioSemi, Amsterdam, Netherlands) served as the EEG amplifier. Prior to data collection, electrode impedances were confirmed to be below 5 kΩ for all channels. EEG data were collected and event-synchronized using OpenViBE^30^, with data acquisition conducted under conditions ensuring less than 2 ms of drift during the recording process. The electrodes were arranged according to the extended international 10–20 system^31^, covering the entire scalp, with the CMS/DRL positioned near the Pz region (see Fig. 1D). The data repository for this study is available at: [10.6084/m9.figshare.28806815.v2]^32^.

### Data structure

**Fig. 2:**
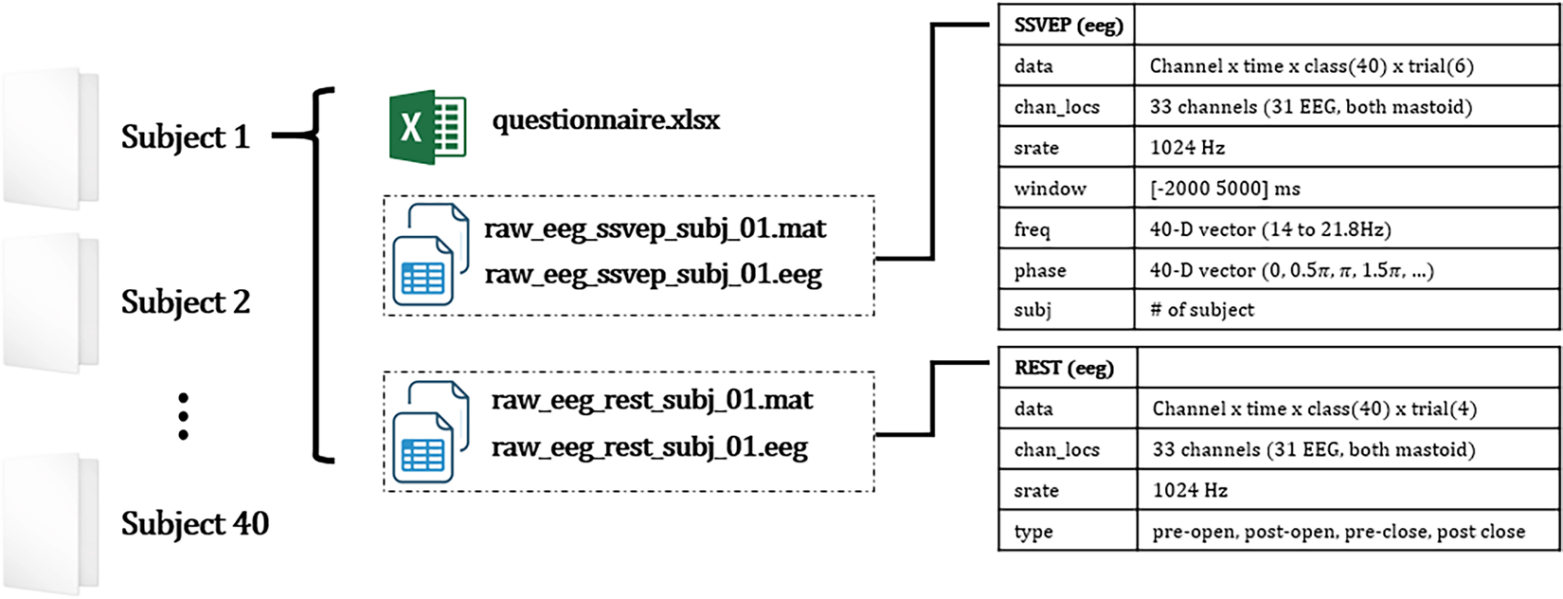
The structure of dataset. Each ‘mat’ file contains the data stored under the variable named ‘eeg’. This ‘eeg’ variable is in MATLAB structure format, with individual fields such as ‘eeg.data’, ‘eeg.chan_locs’, etc., storing the corresponding components of the dataset.

For each subject, the EEG data were organized into two distinct ‘mat’ files corresponding to the Resting State and SSVEP task recordings. The resting state data comprise four 60-second segments: pre-experiment; and eyes open and eyes closed conditions post-experiment. The SSVEP task data are epoched from −2000 ms to 5000 ms relative to the stimulus onset and are organized into 40 classes across 6 blocks, with stimulus parameters stored sequentially (see Fig. 2). Due to a personal issue, post-experiment resting state data for Subject 1 were not recorded; however, this subject successfully completed the pre-experiment resting state recording and fully participated in the SSVEP task. For information regarding data storage and access, please refer to the Code Availability section.

### Questionnaire

In the experimental sequence illustrated in Fig. 1A, participants completed the questionnaires twice: once before and after the experiment. The questionnaire items, detailed in Table 1, were organized as follows: Sections A, B, and C were filled out before the experiment, while Sections D and E were completed afterward. All responses were saved in Excel (.xlsx) format, with each file labeled according to the participant’s number.

## Technical Validation

### Classification methods

#### Standard CCA (stdCCA)

Canonical Correlation Analysis (CCA) is a multivariate statistical method that reveals the underlying correlation between two matrices^33^. CCA calculates the relation between two multi-variable datasets after a linear combination of original data. In most studies, only the first maximum canonical coefficient is employed, and other canonical coefficients are worth considering.

In the context of two multidimensional variables, **X** and **Y**, we are interested in finding linear combinations defined as ${\bf{x}}={{\bf{X}}}^{{\bf{T}}}{{\boldsymbol{\omega }}}_{{\boldsymbol{x}}}$ and ${\bf{y}}={{\bf{Y}}}^{{\bf{T}}}{{\boldsymbol{\omega }}}_{{\boldsymbol{y}}}{\boldsymbol{.}}$ to maximize the correlation between **X** and **Y**. This is achieved by solving the following optimization problem:

$$\mathop{{\bf{\max }}}\limits_{{{\boldsymbol{W}}}_{{\boldsymbol{x}}},{{\boldsymbol{W}}}_{{\boldsymbol{y}}}}{\boldsymbol{\rho }}\left({\boldsymbol{x}},{\boldsymbol{y}}\right)=\frac{{\boldsymbol{E}}[{{\boldsymbol{x}}}^{{\boldsymbol{T}}}{\boldsymbol{y}}]}{\sqrt{{\boldsymbol{E}}\left[{{\boldsymbol{x}}}^{{\boldsymbol{T}}}{\boldsymbol{x}}\right]{\boldsymbol{E}}[{{\boldsymbol{y}}}^{{\boldsymbol{T}}}{\boldsymbol{y}}]}}$$

The maximum value of ***ρ*** with respect to ${{\boldsymbol{\omega }}}_{{\boldsymbol{x}}}\,$ and ${{\boldsymbol{\omega }}}_{{\boldsymbol{y}}}$ represents the maximum canonical correlation. The projections onto ${{\boldsymbol{\omega }}}_{{\boldsymbol{x}}}\,$ and ${{\boldsymbol{\omega }}}_{{\boldsymbol{y}}}$ are known as canonical variates. Here, X denotes the set of multi-channel EEG signals, while **Y** represents the corresponding set of reference signals that share the same length as **X**.

$${{\bf{Y}}}_{{{\boldsymbol{f}}}_{{\boldsymbol{N}}}}=\left[\begin{array}{c}\begin{array}{c}{\bf{\sin }}({\bf{2}}{\boldsymbol{\pi }}{{\boldsymbol{f}}}_{{\boldsymbol{N}}}{\boldsymbol{t}})\\ {\bf{\cos }}({\bf{2}}{\boldsymbol{\pi }}{{\boldsymbol{f}}}_{{\boldsymbol{N}}}{\boldsymbol{t}})\\ \vdots \end{array}\\ {\bf{\sin }}({\bf{2}}{\boldsymbol{\pi }}{{\boldsymbol{N}}}_{{\boldsymbol{h}}}{{\boldsymbol{f}}}_{{\boldsymbol{N}}}{\boldsymbol{t}})\\ {\bf{\cos }}({\bf{2}}{\boldsymbol{\pi }}{{\boldsymbol{N}}}_{{\boldsymbol{h}}}{{\boldsymbol{f}}}_{{\boldsymbol{N}}}{\boldsymbol{t}})\end{array}\right],{\boldsymbol{t}}=\frac{{\bf{1}}}{{{\boldsymbol{f}}}_{{\boldsymbol{s}}}},\frac{{\bf{2}}}{{{\boldsymbol{f}}}_{{\boldsymbol{s}}}},\ldots ,\frac{{\boldsymbol{N}}}{{{\boldsymbol{f}}}_{{\boldsymbol{s}}}}.$$

#### FB-CCA

Filter bank-CCA(FB-CCA) is widely used in feature extraction for SSVEP-based BCI systems. This method makes it difficult to accurately describe the mutative, complex, and different physiological SSVEP signals. Therefore, it is a suitable approach for massive target improvement in classification performance.

Apply a band-pass filter to the EEG signal (**X**) to obtain N sub-bands (${{\bf{X}}}_{{\bf{1}}}\,$ to ${{\bf{X}}}_{{\bf{n}}}$). Then, calculate the following values from the vector composed of the coefficients between each ${{\bf{X}}}_{{\bf{n}}}$ and the reference signal (**Y**). The frequency of the reference signals with the maximal ${{\boldsymbol{\rho }}}_{{\boldsymbol{k}}}$ is considered to be the frequency of the SSVEPs.

$${{\boldsymbol{\rho }}}_{{\boldsymbol{k}}}=\left[\begin{array}{c}{{\boldsymbol{\rho }}}_{{\boldsymbol{k}}}^{{\bf{1}}}\\ {{\boldsymbol{\rho }}}_{{\boldsymbol{k}}}^{{\bf{2}}}\\ \begin{array}{c}{{\boldsymbol{\rho }}}_{{\boldsymbol{k}}}^{{\bf{2}}}\\ \vdots \\ {{\boldsymbol{\rho }}}_{{\boldsymbol{k}}}^{{\boldsymbol{N}}}\end{array}\end{array}\right],\widetilde{{{\boldsymbol{\rho }}}_{{\boldsymbol{k}}}}={\sum {\boldsymbol{w}}\left({\boldsymbol{n}}\right)\ast {({\boldsymbol{\rho }}}_{{\boldsymbol{k}}}^{{\boldsymbol{n}}})}^{{\bf{2}}},{\bf{w}}\left({\bf{n}}\right)={{\boldsymbol{n}}}^{-{\boldsymbol{a}}}+{\boldsymbol{b}},$$

in this study, a = 1, b = 0.

#### IT-CCA

Individual template-CCA(IT-CCA) improves the performance at the expense of additional training with post-stimulus data per subject^34^. One drawback of IT-CCA is excessive reliance on post-stimulus data in the training phase. The combination of CCA and IT-CCA is used for detecting SSVEP signals.

Instead of using the ideal signal (**Y**) as the reference signal, the classification is performed by utilizing an individual template ($\widetilde{{\boldsymbol{X}}}$) as the reference. CCA-based spatial filters (${{\boldsymbol{\omega }}}_{{\boldsymbol{XY}}}$) identify a CCA weight between X and Y.

$${\widetilde{{\boldsymbol{X}}}}_{{\boldsymbol{k}}}=\frac{{\bf{1}}}{{\boldsymbol{N}}}\sum {{\boldsymbol{\chi }}}_{{\boldsymbol{k}}},{\bf{\chi \; is\; training\; trials}},\mathop{{\bf{\max }}}\limits_{{{\boldsymbol{W}}}_{{\boldsymbol{x}}},{{\boldsymbol{W}}}_{\widetilde{{\boldsymbol{x}}}}}{\boldsymbol{\rho }}\left({\boldsymbol{x}},\widetilde{{\boldsymbol{x}}}\right)=\frac{{\boldsymbol{E}}[{{\boldsymbol{x}}}^{{\boldsymbol{T}}}\widetilde{{\boldsymbol{x}}}]}{\sqrt{{\boldsymbol{E}}\left[{{\boldsymbol{x}}}^{{\boldsymbol{T}}}{\boldsymbol{x}}\right]{\boldsymbol{E}}[{\widetilde{{\boldsymbol{x}}}}^{{\boldsymbol{T}}}\widetilde{{\boldsymbol{x}}}]}},\widetilde{{\boldsymbol{x}}}={\widetilde{{\boldsymbol{X}}}}^{{\bf{T}}}{{\boldsymbol{\omega }}}_{\widetilde{{\boldsymbol{x}}}}$$

$${{\boldsymbol{\rho }}}_{{\boldsymbol{k}}}=\left[\begin{array}{c}{\boldsymbol{\rho }}({{\boldsymbol{X}}}^{{\boldsymbol{T}}},\,{{\boldsymbol{Y}}}_{{\boldsymbol{f}}})\\ {\boldsymbol{\rho }}({{\boldsymbol{X}}}^{{\boldsymbol{T}}}{{\boldsymbol{\omega }}}_{{\boldsymbol{X}}\widetilde{{\boldsymbol{X}}}},{\widetilde{{\boldsymbol{X}}}}^{{\boldsymbol{T}}}{{\boldsymbol{\omega }}}_{{\boldsymbol{X}}\widetilde{{\boldsymbol{X}}}})\\ \begin{array}{c}{\boldsymbol{\rho }}({{\boldsymbol{X}}}^{{\boldsymbol{T}}}{{\boldsymbol{\omega }}}_{{\boldsymbol{XY}}},\widetilde{{{\boldsymbol{X}}}^{{\boldsymbol{T}}}}{{\boldsymbol{\omega }}}_{{\boldsymbol{XY}}})\\ {\boldsymbol{\rho }}({{\boldsymbol{X}}}^{{\boldsymbol{T}}}{{\boldsymbol{\omega }}}_{\widetilde{{\boldsymbol{X}}}{\boldsymbol{Y}}},{\widetilde{{\boldsymbol{X}}}}^{{\boldsymbol{T}}}{{\boldsymbol{\omega }}}_{\widetilde{{\boldsymbol{X}}}{\boldsymbol{Y}}})\end{array}\end{array}\right],\widetilde{{\boldsymbol{\rho }}}=\sum {\boldsymbol{sign}}\left({{\boldsymbol{\rho }}}_{{\boldsymbol{i}}}\right)* {{\boldsymbol{\rho }}}_{{\boldsymbol{i}}}^{{\bf{2}}}$$

#### TRCA

Task related component analysis (TRCA) as a user-dependent training method can obtain spatial filters that extract task related source activities from multi-channel EEG signals^35^. It extracts task related components by maximizing their reproducibility during task periods. Therefore, it is assumed that the EEG signal **x(t)** consists of a task-related component ***s(t)*** and unrelated components ***n(t)***, following as:

$${{\bf{x}}}_{{\bf{j}}}\left({\boldsymbol{t}}\right)={{\boldsymbol{a}}}_{{\bf{1}}}{\boldsymbol{s}}\left({\boldsymbol{t}}\right)+{{\boldsymbol{a}}}_{{\bf{2}}}{\boldsymbol{n}}\left({\boldsymbol{t}}\right)$$

$${\bf{y}}\left({\bf{t}}\right)=\sum {{\boldsymbol{\omega }}}_{{\boldsymbol{j}}}{{\boldsymbol{x}}}_{{\boldsymbol{j}}}({\boldsymbol{t}})\;{\bf{and}}\;\sum {{\boldsymbol{\omega }}}_{{\boldsymbol{j}}}{{\boldsymbol{a}}}_{{\bf{1}}{\boldsymbol{j}}}={\bf{1}},\sum {{\boldsymbol{\omega }}}_{{\boldsymbol{j}}}{{\boldsymbol{a}}}_{{\bf{2}}{\boldsymbol{j}}}={\bf{0}}$$

To recover the task-related component ***s(t)***, the inter-trial covariance maximization is calculated as follows:

$$\sum {{\boldsymbol{C}}}_{{\boldsymbol{h}}{\bf{1}},{\boldsymbol{h}}{\bf{2}}}=\sum \sum {{\boldsymbol{\omega }}}_{{\boldsymbol{j}}{\bf{1}}}{{\boldsymbol{\omega }}}_{{\boldsymbol{j}}{\bf{2}}}{\boldsymbol{Cov}}({{\boldsymbol{x}}}_{{\boldsymbol{j}}{\bf{1}}},{{\boldsymbol{x}}}_{{\boldsymbol{j}}{\bf{2}}})={{\boldsymbol{\omega }}}^{{\boldsymbol{T}}}{\boldsymbol{S}}{\boldsymbol{\omega }}$$

$${\bf{Var}}\left({\bf{y}}\left({\bf{t}}\right)\right)=\sum {{\boldsymbol{\omega }}}_{{\boldsymbol{j}}{\bf{1}}}{{\boldsymbol{\omega }}}_{{\boldsymbol{j}}{\bf{2}}}{\boldsymbol{Cov}}({{\boldsymbol{x}}}_{{\boldsymbol{j}}{\bf{1}}},{{\boldsymbol{x}}}_{{\boldsymbol{j}}{\bf{2}}})={{\boldsymbol{\omega }}}^{{\boldsymbol{T}}}{\boldsymbol{Q}}{\boldsymbol{\omega }}$$

The optimal coefficient vector for extracting the task-related component s(t) from EEG is as follows:

$$\hat{{\boldsymbol{\omega }}}=\mathop{{\bf{argmax}}}\limits_{{\boldsymbol{\omega }}}\frac{{{\boldsymbol{\omega }}}^{{\boldsymbol{T}}}{\boldsymbol{S}}{\boldsymbol{\omega }}}{{{\boldsymbol{\omega }}}^{{\boldsymbol{T}}}{\boldsymbol{Q}}{\boldsymbol{\omega }}}$$

Frequency detection is performed using the data extracted as **s(t)** from the individual calibration dataset ${{\boldsymbol{\chi }}}_{{\boldsymbol{n}}}$ as the reference.

$${{\bf{r}}}_{{\bf{n}}}={\boldsymbol{\rho }}({{\boldsymbol{X}}}^{{\boldsymbol{T}}}{{\boldsymbol{\omega }}}_{{\boldsymbol{n}}},{{\boldsymbol{\chi }}}_{{\boldsymbol{n}}}^{{\boldsymbol{T}}}{{\boldsymbol{\omega }}}_{{\boldsymbol{n}}})$$

where, ρ(a, b) indicates the Pearson’s correlation analysis between two signals a and b.

#### EEGNet

EEGNET (CNN-based model) designed specifically to extract both temporal and spatial features, and yields superior performance in BCI applications^36^.

### Preprocessing

To validate the performance of the SSVEP data, we selected 11 electrodes from the occipital to parietal regions (Oz, O1, O2, POz, PO3, PO4, PO7, PO8, Pz, P3, and P4) for optimal classification^24^. EEG signals were segmented into epochs ranging from 0–1000 ms up to 0–3000 ms relative to stimulus onset, and performance was compared for each subject across all 40 classes and 6 sessions.

Different classification methods employed distinct band-pass filter configurations, while a common notch filter at 59–61 Hz was applied to remove power line noise. The filter configurations were as follows:

- stdCCA: Designed to capture the fundamental and second harmonic components ([13–44] Hz).
- FB-CCA: Utilized a filter bank to extract up to fourth harmonic components, with sub-bands set as [13–89], [27–89], [41–89], and [55–89] Hz.
- IT-CCA, TRCA, EEGNet: Configured to include the fundamental up to the fourth harmonic components ([13–89] Hz).

For the training-based methods (IT-CCA, TRCA, and EEGNet), a 6-fold cross-validation was performed for each subject across all 6 sessions, employing a test-to-training ratio of 1:5.

### Information transfer rate (ITR)

The information transfer rate (ITR) in BCI systems quantifies the efficiency of communication between the user and the computer^37^. Both classification accuracy and ITR were calculated and compared as performance metrics, reflecting the speed and accuracy with which the BCI translates user intentions into commands. The ITR is computed as follows:

$${\rm{ITR}}({\rm{bit}}/\min )=\frac{{\log }_{2}N+{Plo}{g}_{2}\left(P\right)+\left(1-P\right){\log }_{2}\left(\frac{1-P}{N-1}\right)}{T/60}$$

where:

- *N* is the number of choices or targets (e.g., the number of classes in the SSVEP speller),
- *P* is the accuracy (probability of correct classification), and
- *T* is the time per trial (in seconds), defined in this study as the sum of the cue duration (0.5 s) and the stimulation time.

### Frequency properties from stimulation

The square pulse-based visual stimulation used in this study elicited SSVEP responses. To confirm that the EEG accurately captured the stimulation frequency, we analyzed the correlation between the EEG signal at the fundamental harmonic component and the corresponding stimulus signal (the square pulse) using the Oz electrode. These analyses were conducted in both the time and frequency domains.

### Effect of visual fatigue

To assess the impact of visual fatigue on EEG recordings during the experiment, we examined the absolute power across five frequency bands: delta (1–4 Hz), theta (4–8 Hz), alpha (8–12 Hz), beta (12–30 Hz), and gamma (30–50 Hz). For each session, the first 30 seconds before the start of the experimental trials were segmented into 5-second epochs using a 1-second sliding window to facilitate detailed calculations of spectral power changes.

### SSVEP Performance Evaluation

**Fig. 3:**
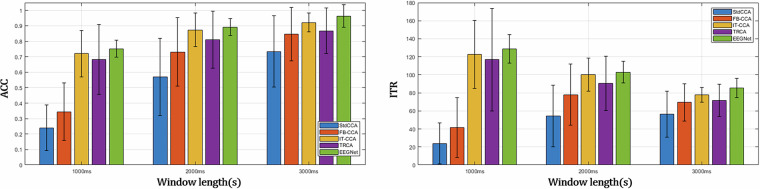
Classification performance and ITR results for each method were evaluated using epoch lengths of 0–1000 ms, 0–2000 ms, and 0–3000 ms. For ITR(bit/min) calculations, the command time was determined as the sum of the cue duration (500 ms) and the respective epoch length.

SSVEP detection performance was evaluated for 40 participants using both classification accuracy and Information Transfer Rate (ITR). Across varying epoch lengths (1000 ms, 2000 ms, and 3000 ms), the classification accuracies(%) were as follows (refer to Fig. 3, left):

- stdCCA: 24.06 ± 14.70%, 56.99 ± 24.90%, 73.44 ± 22.97%
- FB-CCA: 34.44 ± 18.56%, 73.08 ± 22.17%, 84.72 ± 17.26%
- IT-CCA: 72.05 ± 14.95%, 87.42 ± 10.79%, 92.17 ± 6.11%
- TRCA: 68.29 ± 22.73%, 81.08 ± 18.42%, 86.84 ± 14.75%
- EEGNet: 75.23 ± 5.59%, 89.23 ± 6.78%, 96.37 ± 7.28%

The corresponding ITR(bit/min) values are shown in Fig. 3, right:

- stdCCA: 19.67 ± 17.67, 57.12 ± 34.11, 59.26 ± 24.50
- FB-CCA: 41.60 ± 33.14, 77.86 ± 33.88, 69.38 ± 20.62
- IT-CCA: 122.67 ± 37.51, 100.10 ± 18.51, 77.78 ± 8.01
- TRCA: 116.86 ± 56.96, 90.51 ± 30.23, 71.59 ± 17.71
- EEGNet: 128.69 ± 15.77, 102.93 ± 11.90, 85.26 ± 10.60
  Notably, stdCCA achieved the highest ITR with a 3-second window, FB-CCA peaked at a 2-second window, whereas IT-CCA, TRCA, and EEGNet showed optimal performance at a 1-second window. The calibration-based approaches (IT-CCA, TRCA, and EEGNet) demonstrated high-speed detection capabilities, highlighting their potential for efficient, real-time SSVEP-based BCI applications.
  We also evaluated individual performance and assessed cross-subject generalization by validating the IT-CCA and TRCA methods using a leave-one-subject-out (LOSO) cross-validation approach. The performance results are presented below. For more detailed information, please refer to the Supplementary Material 1.
- LOSO templet-CCA: 31.80 ± 18.16%, 63.30 ± 23.97%, 76.47 ± 21.81%
- LOSO with shifting templet CCA: 62.21 ± 26.15%, 79.42 ± 18.78%, 88.22 ± 16.35%
- LOSO TRCA: 65.48 ± 18.10%, 77.01 ± 19.36%, 82.26 ± 20.68%

### Frequency properties from stimulation

**Fig. 4:**
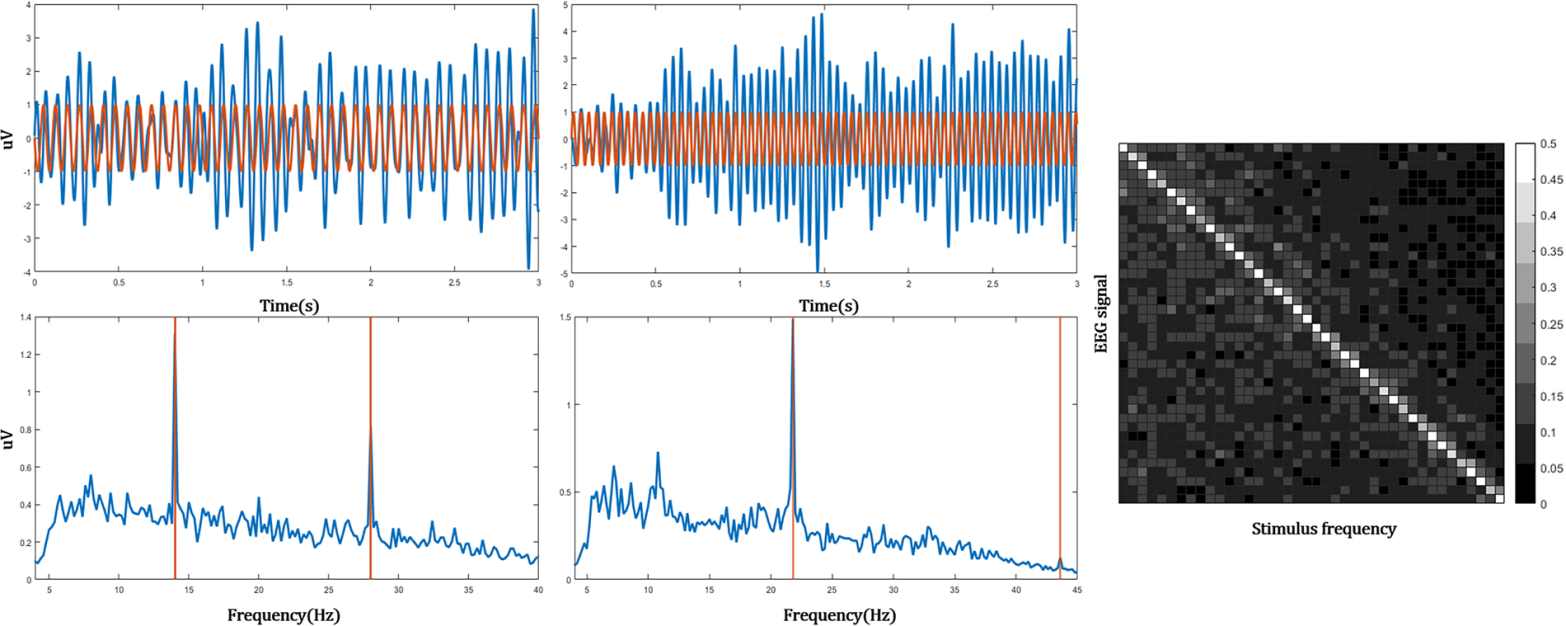
The left panel displays the time-domain and frequency-domain session-averaged EEG signal for Subject 1 at the Oz channel, with frequency analysis based on 14 Hz and 21.8 Hz stimuli over a 0–3000 ms window. The right panel presents the subject-averaged correlation between the stimulus and EEG signals.

To assess the level of SSVEP elicitation in each subject, a band-pass filter (5–45 Hz) was applied to the Oz electrode recordings, capturing up to the second harmonic components. We analyzed the correlation between the EEG signal and the stimulus signal (a square pulse containing the designated frequency) in both the time and frequency domains. The correlation at the stimulation frequency was 0.5124 ± 0.1012, in contrast to 0.0890 ± 0.0446 at non-stimulation frequencies, indicating a significant difference (see Fig. 4).

### Effect of visual fatigue

To investigate the impact of visual fatigue throughout the experiment, we examined session-wise changes in EEG features, focusing on the relative power across five frequency bands: Delta (1–4 Hz), Theta (4–8 Hz), Alpha (8–12 Hz), Beta (12–30 Hz), and Gamma (30–50 Hz). In addition, we analyzed changes in the SSVEP frequency peak, CCA coefficient, and FB-CCA performance across windows ranging from 1000 ms to 5000 ms. To minimize the influence of stimulus-driven responses, the relative power was computed over 30-second intervals during which participants viewed a black screen prior to each session. For detailed analysis, EEG data were segmented into non-overlapping 5-second epochs, and the frequency peak, CCA coefficient, and FB-CCA performance were compared as the window size increased from the onset of stimulation. To further reduce session-dependent calibration effects, classification performance was assessed solely using the calibration-free FB-CCA method Fig. 3.

**Fig. 5:**
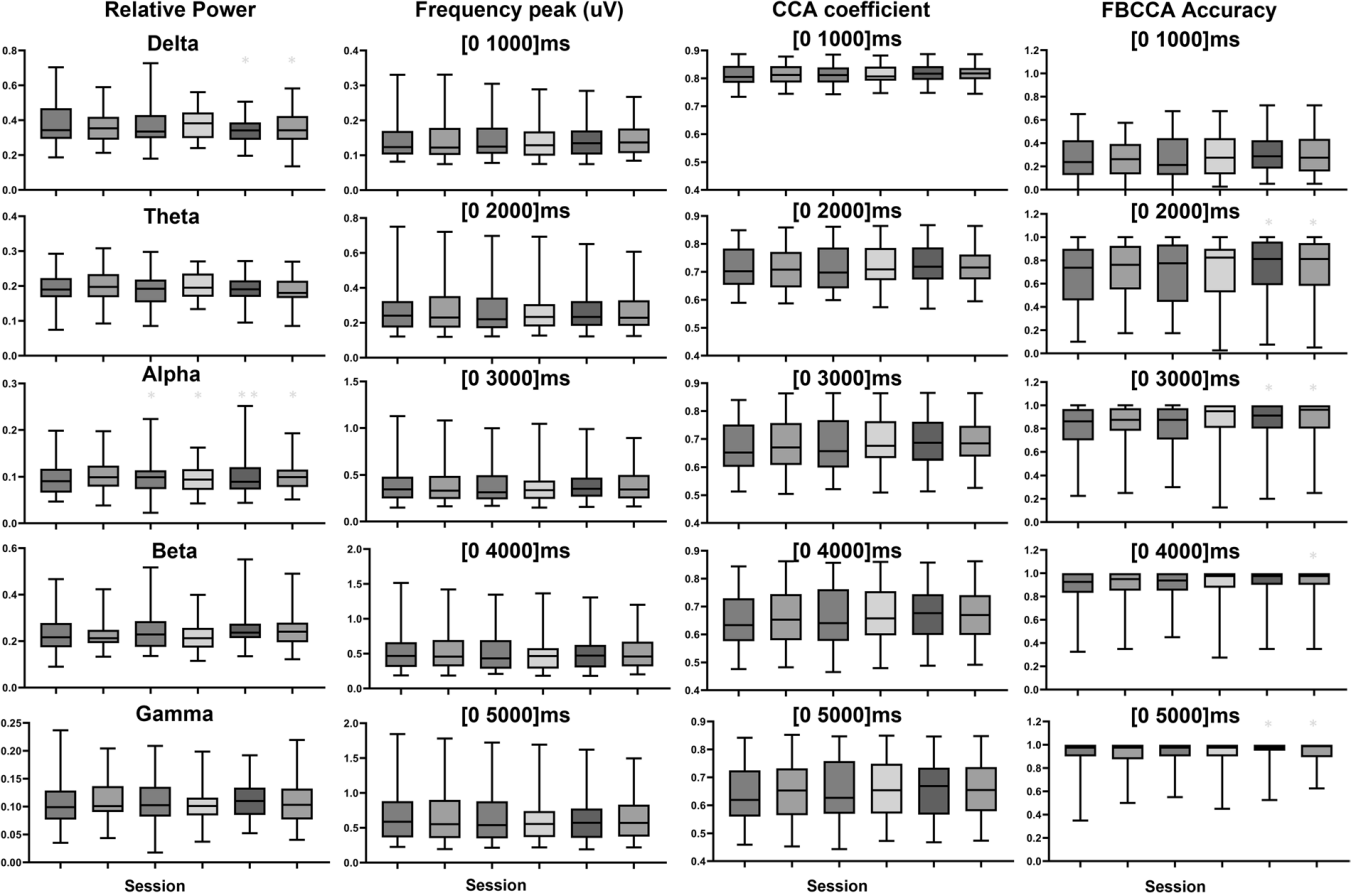
This figure presents relative power, stimulus frequency power, CCA coefficients, and FB-CCA-based classification performance across Sessions 1 to 6. Relative Power; This was calculated across five EEG frequency bands to evaluate spectral changes throughout the sessions. Stimulus Frequency Power; This represents the average peak variations in the SSVEP first harmonic frequency across EEG signals corresponding to each stimulus frequency. CCA Coefficient; This metric measures the correlation between the first harmonic sinusoidal reference and EEG signals across 11 selected channels. Classification Performance; Performance was evaluated using a calibration-free classification method to compare variations in SSVEP decoding across sessions. Statistical comparisons were conducted using paired t-test between Session 1 and other sessions (*p < 0.05, **p < 0.01).

**Fig. 6:**
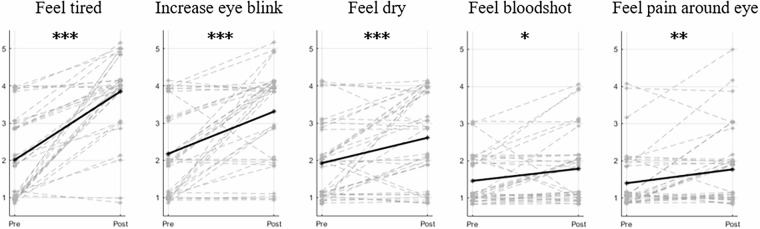
The survey result shows visual fatigue before and after the experiment, related to items C06 to C10 and D06 to D10 in Table 1. The gray lines represent the changes for each subject, while the black line indicates the subject average. Statistical comparisons were conducted using paired t-test (*p < 0.05, **p < 0.01, ***p < 0.005).

A paired t-test, comparing Session 1 with subsequent sessions, revealed statistically significant increases in the relative power of the delta and alpha bands during Sessions 5 and 6 (see Fig. 5). Moreover, classification performance showed a statistically significant improvement in the final session. Changes in visual fatigue were further evaluated through survey items (C06–C10, D06–D10) administered before and after the experiment, with participants reporting significant increases in fatigue, particularly regarding tiredness, eye blinking, and eye dryness (see Fig. 6).

## Supplementary information

Supplementary Material

## Data Availability

All research data has been consented to by participants, with explicit agreement for public sharing. Data sharing follows the appropriate ethical approval procedures to ensure the privacy of participants is fully protected. It is available at https://figshare.com/articles/dataset/SSVEP_40class_dataset/28806815/2.

## Code availability

The GitHub repository [https://github.com/heegyukim4043/SSVEP-dataset-processing] contains the preprocessing scripts, classification models, and survey materials used in this study. While specific model parameters and training procedures are not detailed in this paper, they can be fully replicated using the resources provided in the repository. In our analysis, we utilized MATLAB version 2020a, EEGLAB toolbox version 2022.0, FieldTrip toolbox version 20190618, Python 3.6, and TensorFlow 2.2.0. For detailed information about the requirements for each toolbox and library, please refer to the documentation available in the repository.
