# Supplementary material for: A 40-Class SSVEP Speller Dataset: Beta Range Stimulation for Low-Fatigue BCI Applications
Source: Sci Data. 2025 Nov 5;12:1751. doi: 10.1038/s41597-025-06032-2 (PMC12589495; doi:10.1038/s41597-025-06032-2)
Supplement: Supplementary file 1 — Supplementary Material [file 41597_2025_6032_MOESM1_ESM.docx]

**Supplementary materials**

**Table of contents**

1. IT-CCA LOSO validation
2. TRCA LOSO validation

**1. IT-CCA LOSO validation**

This section outlines the validation of subject-wise performance using IT-CCA to analyze inter-subject variability. In traditional methods, the CCA reference is created by averaging individual trials aligned with the stimulus onset to produce a template for each individual. In the current leave-one-subject-out (LOSO) validation approach, reference templates were similarly constructed by averaging data from all other subjects included in the training set (39 subjects × 6 trials × 40 classes), resulting in class-specific templates.

To address inter-individual variability in SSVEP response latency, latency correction was implemented. This was achieved by estimating the lag between the fundamental harmonic component of the response and the corresponding sine wave of the target frequency using cross-correlation. The performance results after latency adjustment were then compared with the uncorrected outcomes.

The preprocessing for template construction adhered to the same parameters described in the Technical Validation section. For cross-correlation and latency assessment, a band-pass filter was applied to isolate only the fundamental components within the 13–23 Hz frequency range.


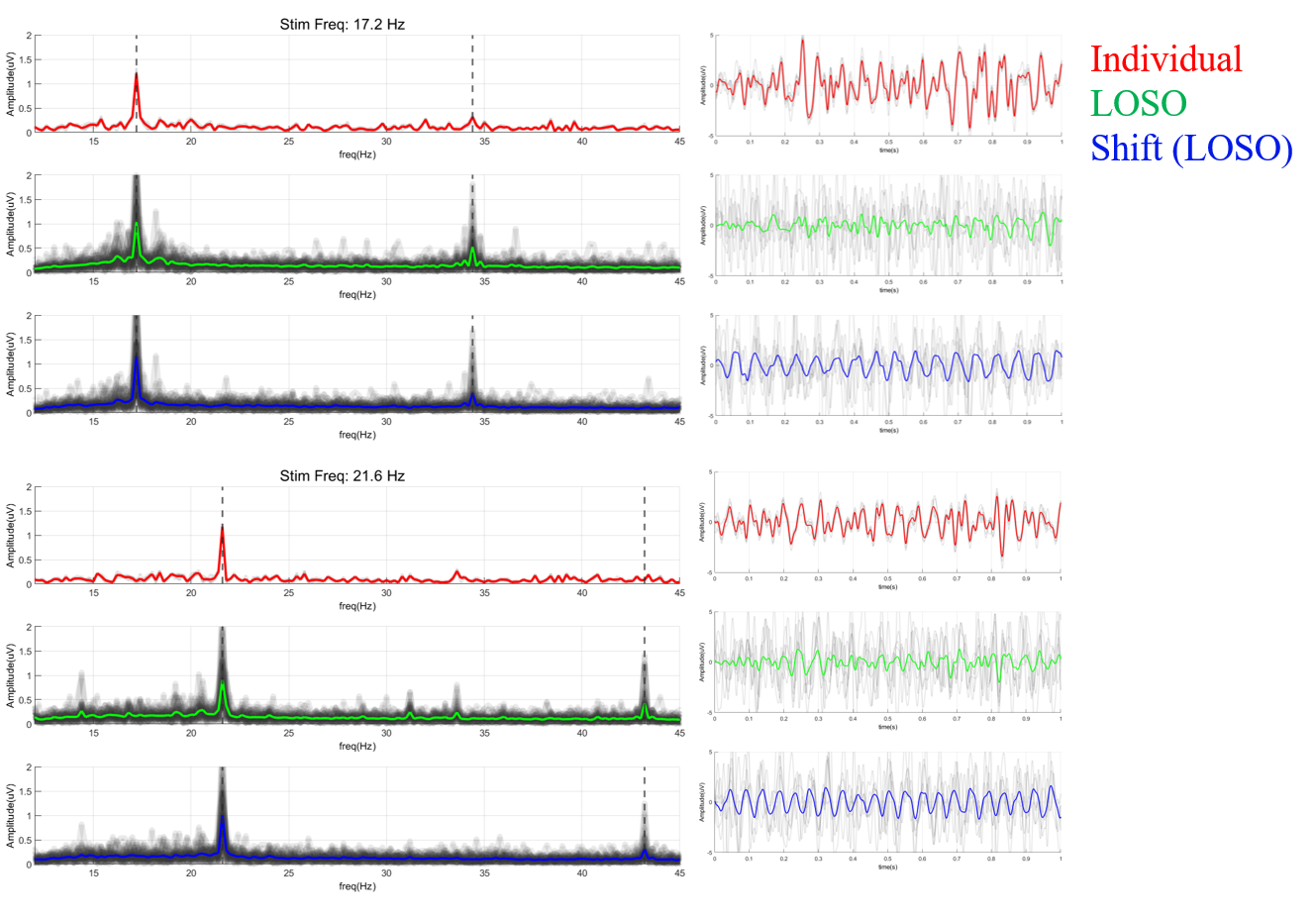


Figure 1. shows the individual template of Subject 1 (red), the LOSO template (green), the LOSO template with latency correction (blue), and each trial used to construct the templates (gray). The LOSO templates were generated using data from Subjects 2 to 40.

As illustrated by the red line in Figure 1, individual responses exhibit similar SSVEP signals, including latency patterns. However, as shown by the green line, the subject-wise template reveals substantial latency differences across individuals, which likely eliminated the characteristic sinusoidal features after averaging and may have contributed to reduced performance. In contrast, as shown by the blue line, applying latency correction based on the fundamental harmonics yielded templates in which the fundamental components were preserved.


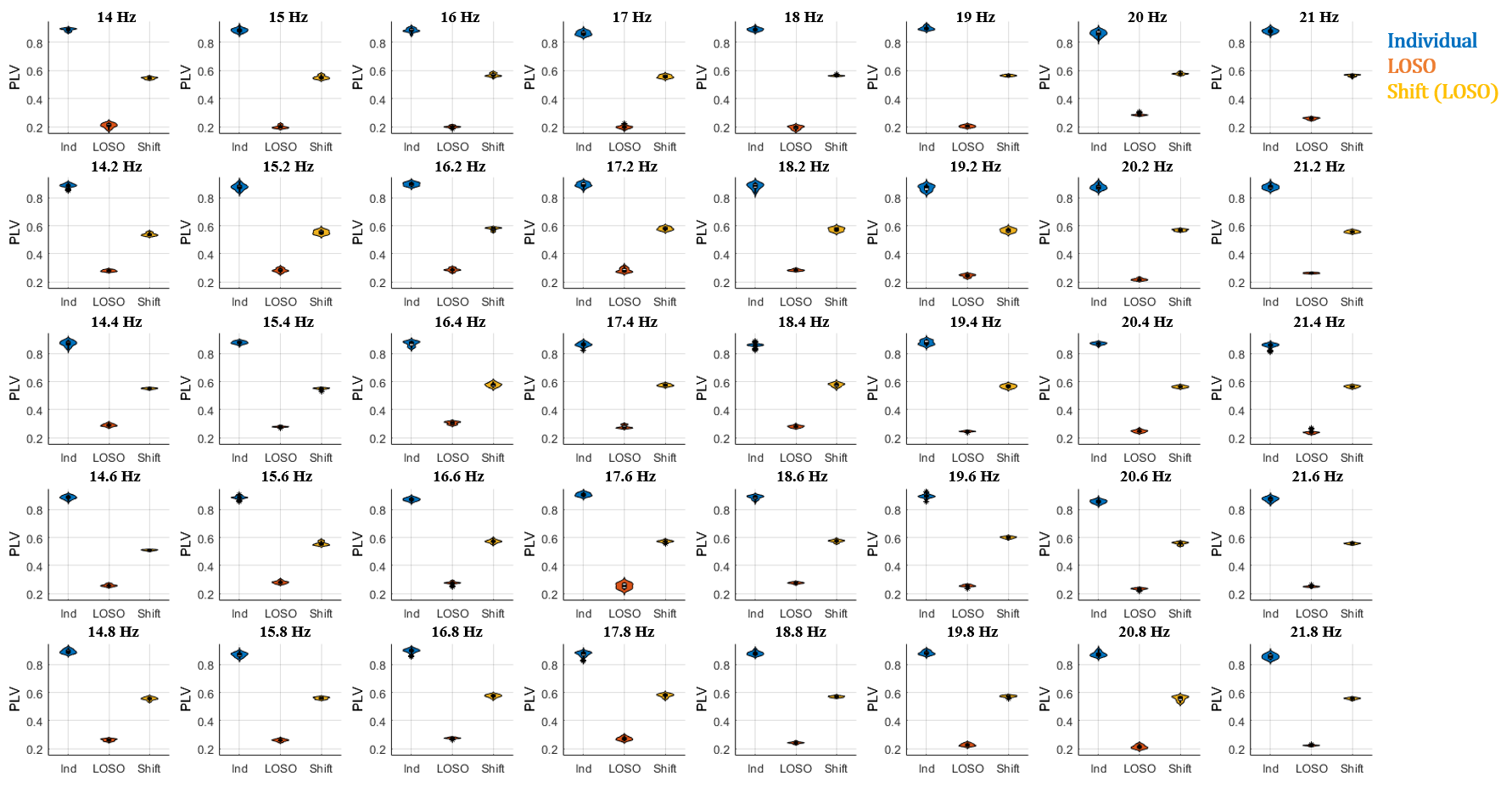


Figure 2. Phase locking value (PLV) between trials used for template construction across 40 subjects, presented separately for each class.

To evaluate latency similarity during template construction, we measured the phase locking value (PLV) between the trials used for each template and compared the results across different classes. As illustrated in Figure 2, individual templates displayed high latency similarity, while the leave-one-subject-out (LOSO) templates demonstrated lower similarity. By applying latency correction through cross-correlation, we were able to enhance this similarity, which was later assessed in terms of performance improvement.


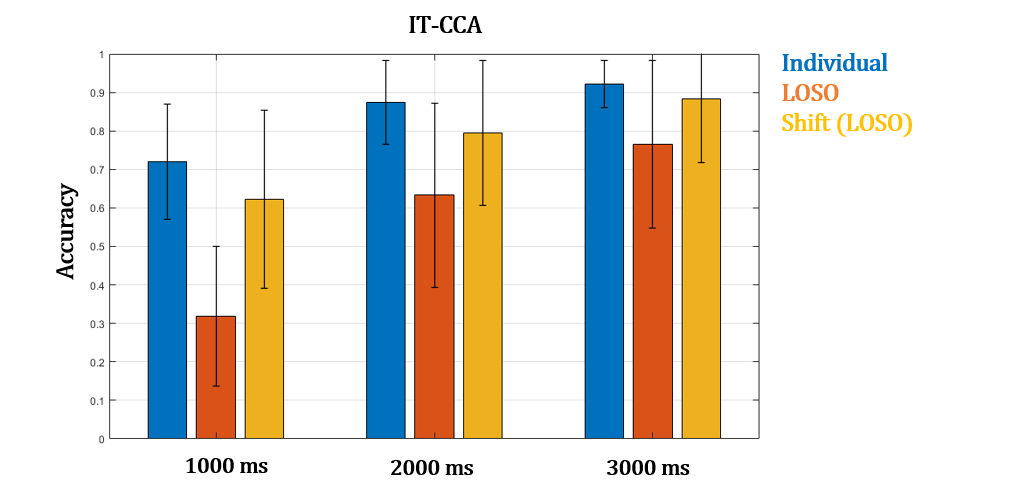


Figure 3. Classification performance (accuracy) of template-based CCA.

The performance comparison between the individual template and the LOSO template are shown below.

- IT-CCA: 72.05 ± 14.95%, 87.42 ± 10.79%, 92.17 ± 6.11%
- LOSO templet-CCA: 31.80 ± 18.16%, 63.30 ± 23.97%, 76.47 ± 21.81%
- LOSO with shifting templet CCA: 62.21 ± 26.15%, 79.42 ± 18.78%, 88.22 ± 16.35%

**2. TRCA LOSO validation**

The second section focuses on validating performance across subjects using Task-Relevant Component Analysis (TRCA) to assess inter-subject variability. In this section, TRCA methodology was applied to derive spatial filters, implementing both individual and LOSO validation approaches as described in the main text. For the LOSO validation, data from other subjects (39 subjects x 6 trials x 40 classes) were utilized for comparison. The preprocessing steps were consistent with those used in the technical validation detailed in the main documents


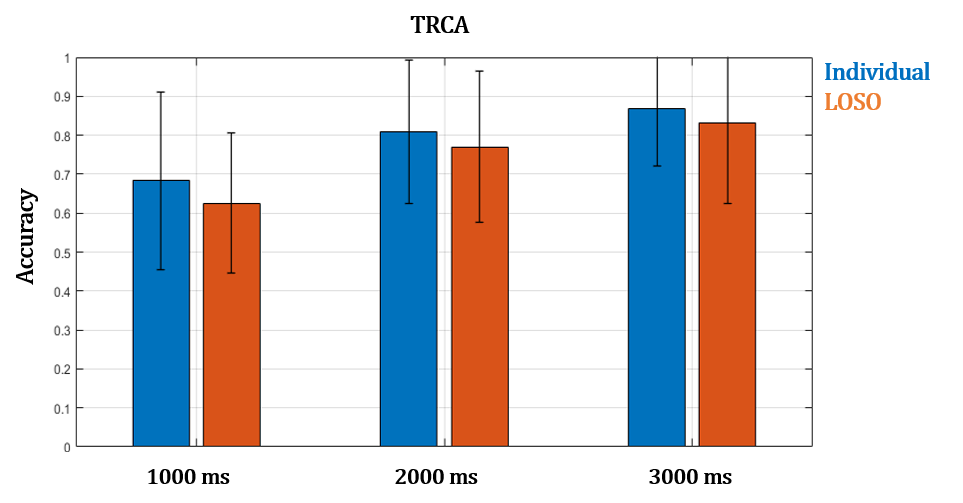


Figure 4. Classification performance (accuracy) of TRCA method.

The performance comparison between the individual filter and the LOSO filter are shown below.

- Individual TRCA: 68.29 ± 22.73%, 81.08 ± 18.42%, 86.84 ± 14.75%
- LOSO TRCA: 65.48 ± 18.10%, 77.01 ± 19.36%, 82.26 ± 20.68%
